# A Fluorescent and Colorimetric Chemosensor for Hg^2+^ Based on Rhodamine 6G With a Two-Step Reaction Mechanism

**DOI:** 10.3389/fchem.2020.00014

**Published:** 2020-02-19

**Authors:** Cui-Bing Bai, Wei-Gang Wang, Jie Zhang, Chang Wang, Rui Qiao, Biao Wei, Lin Zhang, Shui-Sheng Chen, Song Yang

**Affiliations:** ^1^School of Chemistry and Materials Engineering, Fuyang Normal University, Fuyang, China; ^2^Anhui Province Key Laboratory for Degradation and Monitoring of Pollution of the Environment, Fuyang, China

**Keywords:** chemosensor, Hg^2+^, rhodamine 6G, test strips, silica gel plates

## Abstract

A fluorescent and colorimetric chemosensor **L** based on rhodamine 6G was designed, synthesized, and characterized. Based on a two-step reaction, the chemosensor **L** effectively recognized Hg^2+^. The interaction between the chemosensor and Hg^2+^ was confirmed by ultraviolet–visible spectrophotometry, fluorescence spectroscopy, electrospray ionization–mass spectrometry, Fourier-transform infrared spectroscopy, and frontier molecular orbital calculations. The chemosensor **L** was also incorporated into test strips and silica gel plates, which demonstrated good selectivity and high sensitivity for Hg^2+^.

## Introduction

Hg^2+^ is known to be very dangerous to human health because of its extreme toxicity (Chen et al., 2012). Even extremely low levels of Hg^2+^ accumulation in the human body can lead to numerous diseases in human organs (Pan et al., 2018; Peng et al., 2018; Xu et al., 2018). Thus, it is important to develop methods to detect Hg^2+^ with good sensitivity and high selectivity.

Compared to other common methods for Hg^2+^ detection, fluorescent chemosensors have received considerable attention because of their distinct advantages (Nolna and Lippard, 2008; Zhang et al., 2008; Lee et al., 2015; Xie et al., 2015; He et al., 2016; Sakunkaewkasem et al., 2018; Bai et al., 2019; Yuan et al., 2019). As a result, a growing number of fluorescent chemosensors for Hg^2+^ have been reported in the past few decades (Chen et al., 2011; Wang et al., 2012; Park et al., 2013; Cheng et al., 2015; Hong et al., 2016; Lee et al., 2016; Erdemir et al., 2017; Bai et al., 2018; Singh et al., 2018).

**Figure 1:**
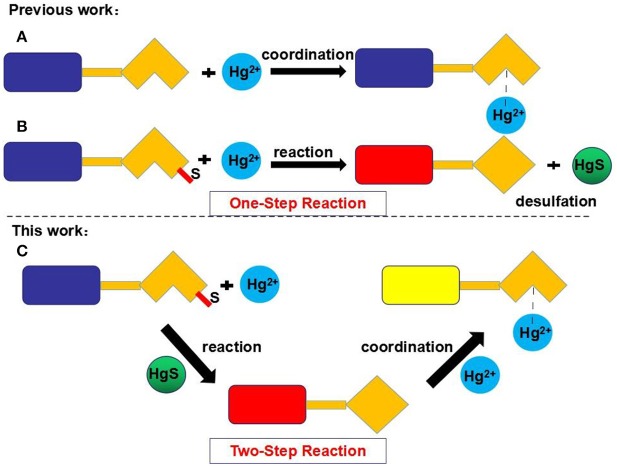
Strategies for the chemosensing of Hg^2+^. Type A: The coordination between ligand and Hg^2+^. Type B: The desulfurization between ligand and Hg^2+^. Type C: The two-step reaction mechanism.

Many reported Hg^2+^ chemosensors were designed in one of two ways based on one-step reactions. In the first type of sensor, the interactions between a ligand and Hg^2+^ results in changes in fluorescence (Figure 1A). The main disadvantage of this type of sensor is its vulnerability to interference from other metal ions, especially Cu^2+^, Cd^2+^, and Fe^3+^ (Wang et al., 2011; Kau et al., 2012; Rao et al., 2012; Zhang et al., 2013; Kim et al., 2014; Fang et al., 2016; Alibert et al., 2017; Huang et al., 2019). In the second type of sensor, special chemical reactions (e.g., desulfurization between the ligands and Hg^2+^) generate a new product with a different fluorescence spectrum (Figure 1B).

In contrast, the fluorescent chemosensor **L** developed in this study recognizes Hg^2+^ through a two-step reaction based on rhodamine 6G. The chemosensor **L** first undergoes a desulfurization reaction with Hg^2+^, and the resulting product then interacts with Hg^2+^ (Figure 1C). As demonstrated in this study, **L** can selectively and sensitively detect Hg^2+^ without interference from other cations, including Cu^2+^, Cd^2+^, and Fe^3+^. Moreover, test strips and silica gel plates based on **L** also exhibit good selectivity for Hg^2+^.

## Materials and Physical Methods

^1^H nuclear magnetic resonance (NMR) and ^13^C NMR spectra were recorded using a Bruker instrument at 400 MHz with tetramethylsilane as the internal standard and deuterated dimethyl sulfoxide (DMSO-d_6_) as the solvent. Ultraviolet–visible (UV–vis) absorption spectra were recorded on a Shimadzu UV-1601 spectrophotometer. Luminescence spectra were recorded on a Horiba Fluoromax-4-NIR spectrometer. Melting points were measured on an X-4 digital melting point apparatus (uncorrected). Infrared (IR) spectra were obtained on a Nicolet 5700 FT-IR spectrophotometer. Fluorescent lifetime and fluorescence quantum yield were measured using a Horiba Fluoromax-4 spectrometer. For fluorescent lifetime measurements, the fluorescence signals were collimated and focused onto the entrance slit of a monochromator, and the output plane was equipped with a photomultiplier tube. The fluorescent decay was then analyzed by least-squares regression, and the quality of the exponential fit was evaluated by the goodness of fit (χ^2^). Mass spectra were recorded with a Shimadzu LCMS-IT/TOF mass spectrometer. All reagents used were of analytical grade.

### Synthesis of Compound L

**Figure 2:**
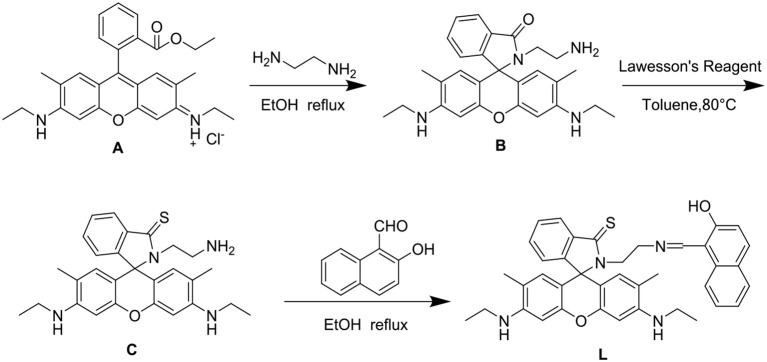
Synthesis of chemosensor **L**. **(A)** Compound **A**. **(B)** Compound **B**. **(C)** Compound **C**.

The synthesis of **L** is outlined in Figure 2. Compound **C** was synthesized according to the reported procedure (Hong et al., 2016). Compound **C** (0.5 mmol) and 2-hydroxy-1-naphthaldehyde (0.5 mmol) were then dissolved in 20 ml of ethanol and refluxed for 12 h with stirring, forming an orange precipitate. The crude product was filtered and purified by silica gel chromatography (ethyl acetate:petroleum ether = 3:1) to obtain the fluorescent chemosensor **L**. Yield 81%, m.p. >300°C, ^1^H NMR (see Figure S1) (400 MHz, DMSO-d_6_, δ): 13.84 (s, 1H), 8.76 (d, *J* = 8.7 Hz, 1H), 7.98 (dd, *J* = 5.4, 3.2 Hz, 1H), 7.92 (d, *J* = 8.4 Hz, 1H), 7.72 (d, *J* = 9.3 Hz, 1H), 7.64 (d, *J* = 7.5 Hz, 1H), 7.59 (dd, *J* = 6.0, 2.6 Hz, 2H), 7.42 (t, *J* = 7.2 Hz, 1H), 7.21 (t, *J* = 7.3 Hz, 1H), 7.07 (dd, *J* = 5.3, 3.0 Hz, 1H), 6.72 (d, *J* = 9.3 Hz, 1H), 6.32 (s, 2H), 6.01 (s, 2H), 5.12 (s, 2H), 3.76 (t, *J* = 6.7 Hz, 2H), 3.12 (dd, *J* = 9.2, 5.8 Hz, 4H), 1.81 (s, 6H), 1.20 (t, *J* = 7.1 Hz, 6H). ^13^C NMR (see Figure S2) (100 MHz, DMSO) δ 191.24, 175.39, 159.96, 151.58, 151.49, 148.69, 137.52, 137.11, 134.29, 133.46, 129.31, 129.25, 128.26, 127.65, 125.93, 125.03, 124.94, 123.77, 122.80, 119.19, 119.00, 106.77, 103.12, 96.17, 73.51, 49.53, 44.63, 37.92, 17.53, 14.56. HRMS (**ESI**, see Figure S3) *m*/*z*: [M+H]^+^ Calcd for C_39_H_39_N_4_O_2_S: 627.2788; Found 627.2786.

### General Spectroscopic Methods

A solution of metal ions was prepared from the nitrate salts of K^+^, Na^+^, Ag^+^, Cu^2+^, Co^2+^, Ca^2+^, Cd^2+^, Mg^2+^, Ba^2+^, Pb^2+^, Fe^2+^, Ni^2+^, Zn^2+^, Mn^2+^, Hg^2+^, Al^3+^, Y^3+^, Ce^3+^, and Fe^3+^ (China Pharmaceutical Co. Ltd., used without further purification). The ligand concentration was kept constant at 1.0 × 10^−5^ M during spectral measurements. The probe solution was prepared in HEPES buffer (10 mM, pH 7.4)/CH_3_CN (40:60, V/V).

## Results and Discussion

**Figure 3:**
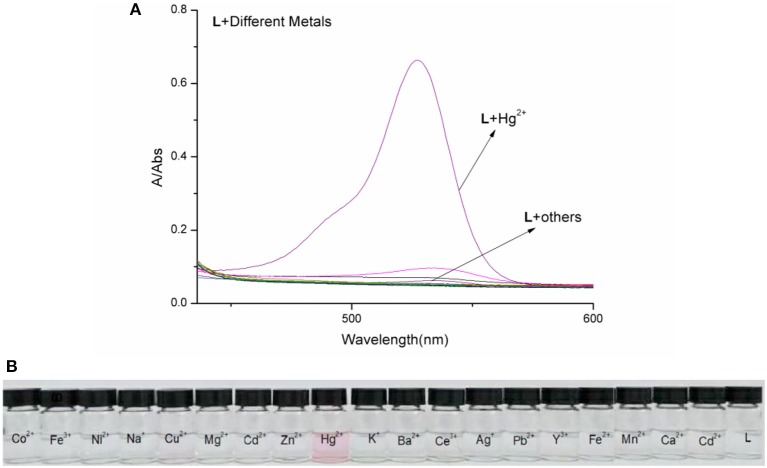
**(A)** Absorption spectra of **L** (1.0 × 10^−5^ M) in the presence of various metal ions in HEPES buffer. **(B)** Photographs of **L** (1.0 × 10^−5^ M) in the presence of various metal ions.

**Figure 4:**
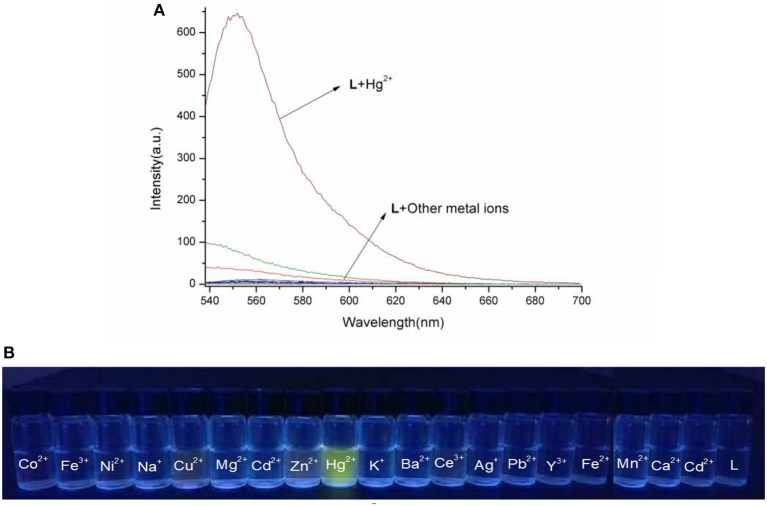
**(A)** Fluorescence spectra of **L** (1.0 × 10^−5^ M) in the presence of various metal ions in HEPES buffer (λ_ex_ = 526 nm). **(B)** Photographs of chemosensor **L** (1.0 × 10^−5^ M) in the presence of various metal ions under the hand-held ultraviolet lamp.

The spectral properties of **L** were studied in HEPES buffer (Figures 3, 4). When the concentration of Hg^2+^ increased to 10 equivalents, a new absorption peak at 526 nm appeared (Figure 3). In the fluorescence spectrum, an emission peak appeared at 550 nm upon excitation at 526 nm (Figure 4). The other cations did not result in the same spectral changes as Hg^2+^. Meanwhile, the color of the solution also changed significantly when 10 equivalents of Hg^2+^ was added to **L**. This color change could be observed directly by the naked eye.

Based on competition experiments, the detection of Hg^2+^ by **L** was not affected by the presence of other cations, including Cu^2+^, Cd^2+^, and Fe^3+^ (Figures S4, S5, **ESI**). Surprisingly, the spectral properties depended on the ratio of [Hg^2+^] to [**L**]. When the ratio did not exceed 1, the spectra did not change markedly before or after Hg^2+^ was added into the solution of **L**. The emission peak at 550 nm did not appear (Figures S6, S7, **ESI**), while no change was observed in the UV–vis spectrum. The fluorescence intensity at 550 nm increased strongly when the ratio exceeded 1, and a new absorption peak at 526 nm appeared. These results are unusual in comparison to previously reported chemosensors for Hg^2+^ (Figures 3, 4).

**Figure 5:**
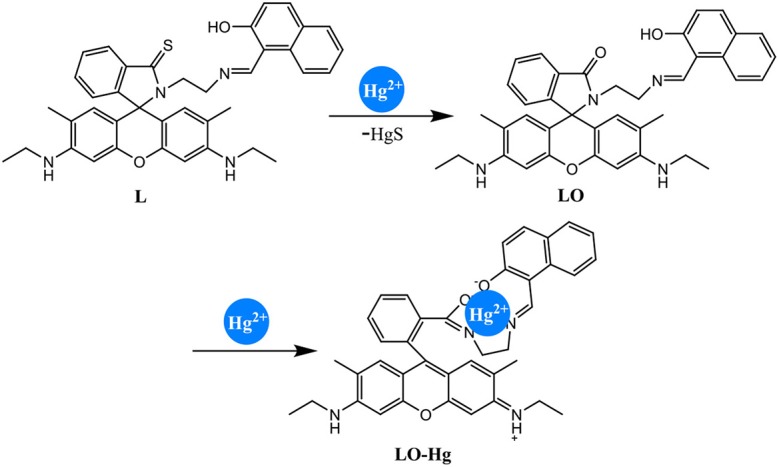
Proposed mechanism for Hg^2+^ detection by chemosensor **L**.

To clarify the unusual results, electrospray ionization–mass spectrometry (ESI-MS) was conducted with different ratios of [Hg^2+^] to [**L**]. When the ratio did not exceed 1, the ion peak was detected at 611.3018 (*m*/*z*), corresponding to the desulfurization reaction product **LO** (Figure S8, **ESI**). This implies that **L** was transformed to **LO** under the action of Hg^2+^ provided that the ratio of [Hg^2+^] to [**L**] did not exceed 1 (Figure 5). Under this condition, Hg^2+^ did not lead to the spirolactam ring opening of **L**. Thus, the fluorescent emission peak at 550 nm did not appear, nor did the absorption peak at 526 nm. Meanwhile, no color change of the solution was observed by the naked eye. The first desulfurization step was completed when [Hg^2+^]:[**L**] = 1. When the ratio exceeded 1, the ion peak at *m*/*z* 813.2609 was classified as [**LO**–Hg^2+^+H^+^]^+^ (Figure S9, **ESI**). Under these conditions, Hg^2+^ caused spirolactam ring opening, generating the emission peak at 550 nm and the absorption peak at 526 nm. In addition, the solution color changed from colorless to pink. After the completion of the first step, Hg^2+^ transformed the closed spirolactam structure into a ring-opened amide in the second step.

The IR spectra support the above two-step reaction mechanism (Figure S10, **ESI**). The peak at 1,153 cm^−1^, which was assigned to the stretching vibration of C=S, disappeared during the first step. The peak at 1,518 cm^−1^, corresponding to the stretching vibration of C=O, vanished in the second step. Therefore, **L** detected Hg^2+^ through a two-step reaction (Figure 5).

**Figure 6:**
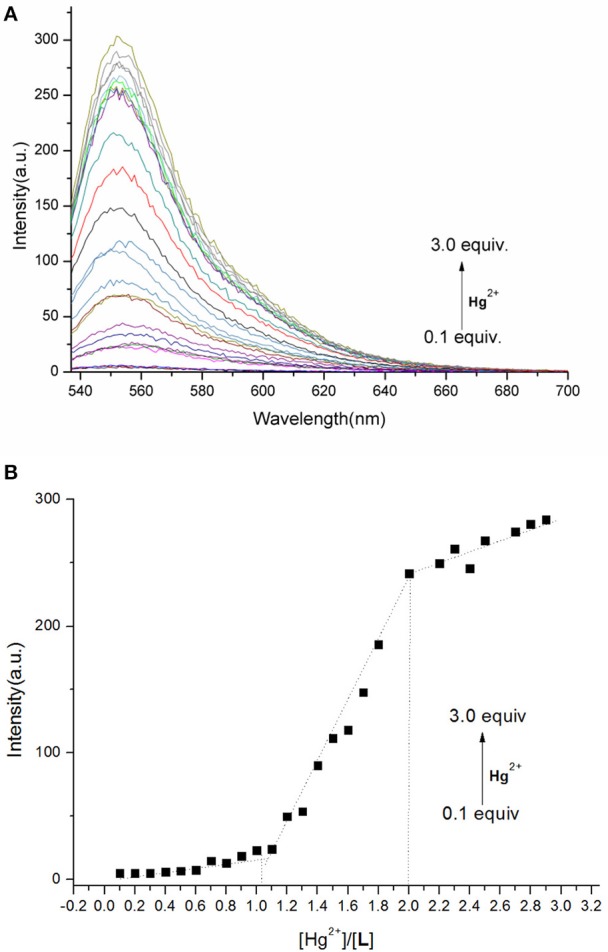
**(A)** Fluorescence spectra of **L** (1.0 × 10^−5^ M) in the presence of different concentrations of Hg^2+^ in HEPES buffer (λ_ex_ = 526 nm). **(B)** Plot of fluorescence intensity at 550 nm vs. Hg^2+^ concentration in the range of 0.1–3.0 equiv.

To understand the interaction between the chemosensor **L** and Hg^2+^, a titration experiment was carried out. The fluorescent intensity at 550 nm hardly changed when [Hg^2+^]:[**L**] changed from 0 to 1, and the absorption spectrum of **L** did not change significantly. However, the fluorescent intensity increased sharply when [Hg^2+^]:[**L**] changed from 1 to 2, and the intensity of the new absorption peak at 526 nm increased greatly. As shown in Figure 3, two inflection points were observed in the absorption spectrum of **L** + Hg^2+^, indicating that **L** might interact with Hg^2+^ in two steps. The stoichiometric ratio between **L** and Hg^2+^ was 1:1 or 1:2. The above results are in accordance with the Job plot (Figure 6 and Figures S11–S13, **ESI**).

**Figure 7:**
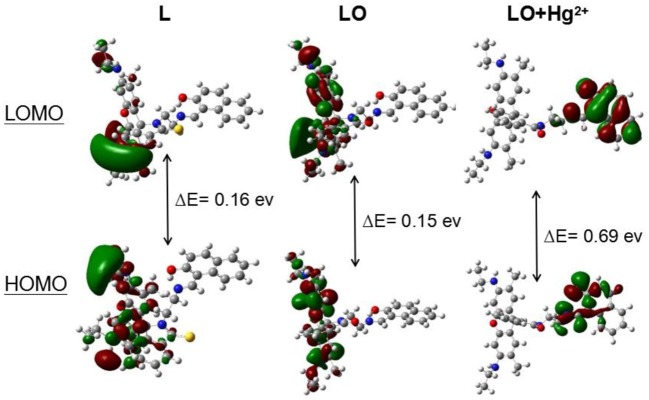
Molecular orbital plots (VESTA software) of the HOMOs and LUMOs of **L**, **LO**, and **LO**–Hg^2+^.

The calculated frontier molecular orbitals and energy levels of **L**, **LO**, and **LO**–Hg^2+^ confirmed the interaction between **L** and Hg^2+^. As shown in Figure 7, the molecular orbital structure of **L** was clearly different from those of **LO** and **LO**–Hg^2+^. The energy gaps between the highest occupied molecular orbital (HOMO) and lowest unoccupied molecular orbital (LUMO) in **L**, **LO**, and **LO**–Hg^2+^ were calculated to be 0.16, 0.15, and 0.69 eV, respectively. The results indicate that Hg^2+^ and **L** were combined via a two-step reaction, which effectively decreased the energy gap and stabilized the system.

The detection limit of **L** toward Hg^2+^ was determined to be 0.012 × 10^−7^ M (Figure S14, **ESI**). Moreover, the fluorescence lifetime and fluorescence quantum yield of **L**–**Hg**^**2+**^ were measured to study the fluorescent properties of **L** (Table S1, **ESI**).

**Figure 8:**
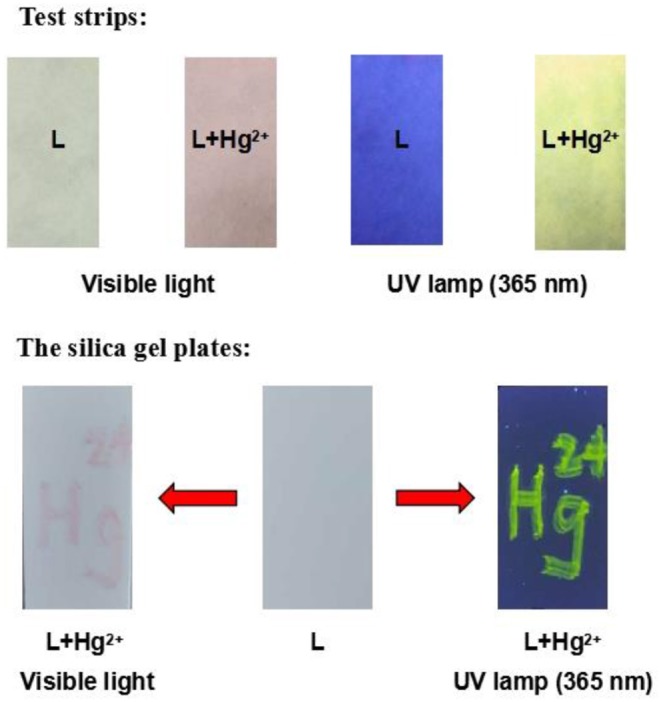
Photographs of test strips and silica gel plates.

To evaluate the practical application potential of the chemosensor **L**, test strips and silica gel plates were prepared using **L**. When Hg^2+^ was added to the test strips, a clear change in color from colorless to pink was observed, and the fluorescence was enhanced (Figure 8). In addition, when “Hg^2+^” was written on the silica gel plate using Hg^2+^ solution, the silica gel plate underwent significant changes. The results indicate that **L** can be used to create smart materials for the detection of Hg^2+^ in aqueous solution.

## Conclusions

In summary, a chemosensor **L** based on rhodamine 6G was designed, synthesized, and characterized. **L** was demonstrated to sensitively and selectively recognize Hg^2+^ via a two-step reaction, different from the one-step mechanisms by which reported chemosensors interact with Hg^2+^. The interaction between **L** and Hg^2+^ was confirmed by UV–vis spectrophotometry, fluorescence spectroscopy, ESI-MS, IR spectroscopy, and frontier molecular orbital calculations. In addition, **L** could be incorporated into test strips and silica gel plates to effectively detect Hg^2+^.

## Data Availability Statement

All datasets generated for this study are included in the article/Supplementary Material.

## Author Contributions

C-BB and RQ designed the work and wrote the manuscript. C-BB, W-GW, JZ, BW, and LZ carried out the experiments. CW, S-SC, and SY performed the spectroscopic experiments. RQ revised and edited the manuscript. All authors reviewed the manuscript and have agreed to its publication.

### Conflict of Interest

The authors declare that the research was conducted in the absence of any commercial or financial relationships that could be construed as a potential conflict of interest.

## References

[B1] Alibert A. Vaiano P. Caporale A. Consales M. Ruvo M. Cusano A. (2017). Fluorescent chemosensors for Hg^2+^ detection in aqueous environment. Sens. Actuators B Chem. 247, 727–735. 10.1016/j.snb.2017.03.026

[B2] Bai C. B. Fan H. Y. Qiao R. Wang S. N. Wei B. Meng Q.. (2019). Synthesis of methionine methyl ester-modified coumarin as the fluorescent-colorimetric chemosensor for selective detection Cu^2+^ with application in molecular logic gate. Spectrochim. Acta. A 216, 45–51. 10.1016/j.saa.2019.03.016 30877893

[B3] Bai C. B. Qiao R. Liao J. X. Xiong W. Z. Zhang J. Chen S. S.. (2018). A highly selective and reversible fluorescence “off-on-off” chemosensor for Hg^2+^ based on rhodamine-6G dyes derivative and its application as a molecular logic gate. Spectrochim. Acta. A 202, 252–259. 10.1016/j.saa.2018.05.050 29800888

[B4] Chen X. Pradhan T. Wang F. Kim J. S. Yoon J. (2011). Fluorescent chemosensors based on spiroring-opening of xanthenes and related derivatives. Chem. Rev. 112, 1910–1956. 10.1021/cr200201z 22040233

[B5] Chen Y. Zhu C. Yang Z. Li J. Jiao Y. He W.. (2012). A new “turn-on” chemodosimeter for Hg^2+^: ICT fluorophore formation via Hg^2+^-induced carbaldehyde recovery from 1, 3-dithiane. Chem. Commun. 48, 5094–5096. 10.1039/c2cc31217d 22514021

[B6] Cheng H. Z. Li G. Liu M. (2015). A metal-enhanced fluorescence sensing platform based on new mercapto rhodamine derivatives for reversible Hg^2+^ detection. J. Hazard. Mater. 287, 402–411. 10.1016/j.jhazmat.2015.01.046 25679802

[B7] Erdemir S. Yuksekogul M. Karakurt S. Kocyigit O. (2017). Dual-channel fluorescent probe based on bisphenol a-rhodamine for Zn^2+^ and Hg^2+^ through different signaling mechanisms and its bioimaging studies. Sens. Actuators B Chem. 241, 230–238. 10.1016/j.snb.2016.10.082

[B8] Fang Y. W. Zhang B. G. Chen J. Kong L. Yang L. M. Bi H. (2016). An aie active probe for specific sensing of Hg^2+^ based on linear conjugated bis-schiff base. Sens. Actuators B Chem. 229, 338–346. 10.1016/j.snb.2016.01.130

[B9] He W. L. Dong L. B. Liu Y. Lin Y. W. (2016). Fluorescent chemosensors manipulated by dual/triple interplaying sensing mechanisms. Chem. Soc. Rev. 45, 6449–6461. 10.1039/C6CS00413J 27711651

[B10] Hong M. Lu X. Chen Y. Xu D. (2016). A novel rhodamine-based colorimetric and fluorescent sensor for Hg^2+^ in water matrix and living cell. Sens. Actuators B Chem. 232, 28–36. 10.1016/j.snb.2016.03.125

[B11] Huang L. Yang Z. Zhou Z. Li Y. Tang S. Xiao W. (2019). A dual colorimetric and near-infrared fluorescent turn-on probe for Hg^2+^ detection and its applications. Dyes Pigments 163, 118–125. 10.1016/j.dyepig.2018.11.047

[B12] Kau P. Kau S. Singh K. (2012). Bis (N-methylindolyl). methane-based chemical probes for Hg^2+^ and Cu^2+^ and molecular implication gate operating in fluorescence mode. Org. Biomol. Chem. 10, 1497–1501. 10.1039/c2ob06793e 22228473

[B13] Kim I. Lee E. N. Jeong J. Y. Chung H. Y. Cho K. B. Lee E. (2014). Micellar and vesicular nanoassemblies of triazole-based amphiphilic probes triggered by mercury, (II). ions in a 100% aqueous medium. Chem. Commun. 50, 14006–14009. 10.1039/C4CC06742H 25266767

[B14] Lee J. J. Kim S. Y. Nam E. Lee Y. S. Lim H. M. Kim C. (2016). A PET-based fluorometric chemosensor for the determination of mercury (II) and pH, and hydrolysis reaction-based colorimetric detection of hydrogen sulfide. Dalton Trans. 45, 5700–5712. 10.1039/C6DT00147E 26928649

[B15] Lee M. Kim S. J. Sessler L. J. (2015). Small molecule-based ratiometric fluorescence probes for cations, anions, and biomolecules. Chem. Soc. Rev. 44, 4185–4191. 10.1039/C4CS00280F 25286013 PMC4387118

[B16] Nolna M. E. Lippard J. S. (2008). Tools and tactics for the optical detection of mercuric ion. Chem. Rev. 108, 3443–3480. 10.1021/cr068000q 18652512

[B17] Pan L. S. Li K. Li L. L. Li Y. M. Shi L. Liu H. Y. (2018). A reaction-based ratiometric fluorescent sensor for the detection of Hg(II) ions in both cells and bacteria. Chem. Commun. 54, 4955–4958. 10.1039/C8CC01031E 29701217

[B18] Park S. Kim W. Swamy K. M. K. Lee H. Y. Jung J. Y. Kim G. (2013). Rhodamine hydrazone derivatives bearing thiophene group as fluorescent chemosensors for Hg^2+^. Dyes Pigments 99, 323–328. 10.1016/j.dyepig.2013.05.015

[B19] Peng D. Zhang L. Liang P. R. Qiu D. J. (2018). Rapid detection of mercury ions based on nitrogen-doped graphene quantum dots accelerating formation of manganese porphyrin. ACS Sens. 3, 1040–1047. 10.1021/acssensors.8b00203 29667403

[B20] Rao S. A. Kim D. Y. Wang J. T. Kim H. K. Wang H. S. Ahn H. K. (2012). Reaction-based two-photon probes for mercury ions: fluorescence imaging with dual optical windows. Org. Lett. 14, 2598–2601. 10.1021/ol3009057 22564078

[B21] Sakunkaewkasem S. Petdum A. Panchan W. Sirirak J. Charoenpanich A. Sooksimuang T.. (2018). Dual-analyte fluorescent sensor based on [5] helicene derivative with super large stokes shift for the selective determinations of Cu^2+^ or Zn^2+^ in buffer solutions and its application in a living cell. ACS Sens. 3, 1016–1023. 10.1021/acssensors.8b00158 29733581

[B22] Singh G. Ran S. Sharmai G. Kalra P. Singh N. Verma V. (2018). Coumarin-derived organosilatranes: functionalization at magnetic silica surface and selective recognition of Hg^2+^ ion. Sens. Actuators B Chem. 266, 861–872. 10.1016/j.snb.2018.03.036

[B23] Wang F. Nam S. W. Guo Z. Park S. Yoon J. (2012). A new rhodamine derivative bearing benzothiazole and thiocarbonyl moieties as a highly selective fluorescent and colorimetric chemodosimeter for Hg^2+^. Sens. Actuators B Chem. 161, 948–953. 10.1016/j.snb.2011.11.070

[B24] Wang Y. Huang Y. Li B. Zhang L. Song H. Jiang H. (2011). A cell compatible fluorescent chemosensor for Hg^2+^ based on a novel rhodamine derivative that works as a molecular keypad lock. RSC Adv. 1, 1294–1300. 10.1039/c1ra00488c

[B25] Xie H. P. Guo Q. F. Wang Y. L. Yang S. Yao H. D. Yang Y. G. (2015). A dansyl-rhodamine ratiometric fluorescent probe for Hg^2+^ based on fret mechanism. J. Fluoresc. 25, 319–325. 10.1007/s10895-015-1511-7 25597044

[B26] Xu D. Yu S. Yin Y. Wang S. Lin Q. Yuan Z. (2018). Sensitive colorimetric Hg^2+^ detection via amalgamation-mediated shape transition of gold nanostars. Front. Chem. 6:566. 10.3389/fchem.2018.00566 30538981 PMC6277514

[B27] Yuan X. Leng H. T. Guo Q. Z. Wang Y. C. Li Z. J. Yang W. W. (2019). A fret-based dual-channel turn-on fluorescence probe for the detection of Hg^2+^ in living cells. Dyes Pigments 161, 403–410. 10.1016/j.dyepig.2018.09.078

[B28] Zhang P. Shi B. B. Zhang Y. M. Lin Q. Yao H. You X. M. (2013). A selective fluorogenic chemodosimeter for Hg^2+^ based on the dimerization of desulfurized product. Tetrahedron 69, 10292–10298. 10.1016/j.tet.2013.10.024

[B29] Zhang X. Xiao Y. Qian X. (2008). A ratiometric fluorescent probe based on fret for imaging Hg^2+^ ions in living cells. Angew. Chem. Int. Ed. 47, 8025–8029. 10.1002/anie.200803246 18792904

