# Supplementary material for: A Fluorescent and Colorimetric Chemosensor for Hg2+ Based on Rhodamine 6G With a Two-Step Reaction Mechanism
Source: Front Chem. 2020 Feb 19;8:14. doi: 10.3389/fchem.2020.00014 (PMC7042397; doi:10.3389/fchem.2020.00014)
Supplement: Supplementary file 1 [file Data_Sheet_1.docx]

Supplementary Material

A fluorescent and colorimetric chemosensor for Hg^2+^ based on rhodamine 6G with a two-step reaction mechanism

Cui-Bing Bai^1,2^, Wei-Gang Wang^1^, Jie Zhang^1^, Chang Wang^1,2^, Rui Qiao^1,2*^, Biao Wei^1,2^, Lin Zhang^1,2^, Shui-Sheng Chen^1,2^, Song Yang^1,2^

*^1^School of Chemistry and Materials Engineering, Fuyang Normal University, Fuyang, Anhui Province, China*

*^2^Anhui Province Key Laboratory for Degradation and Monitoring of Pollution of the Environment, Fuyang, Anhui Province, China*

*Corresponding Author

Rui Qiao, [qiaorui@mail.ipc.ac.cn](mailto:qiaorui@mail.ipc.ac.cn) Tel: +86-558-2595626; Fax: +86-558-2596249


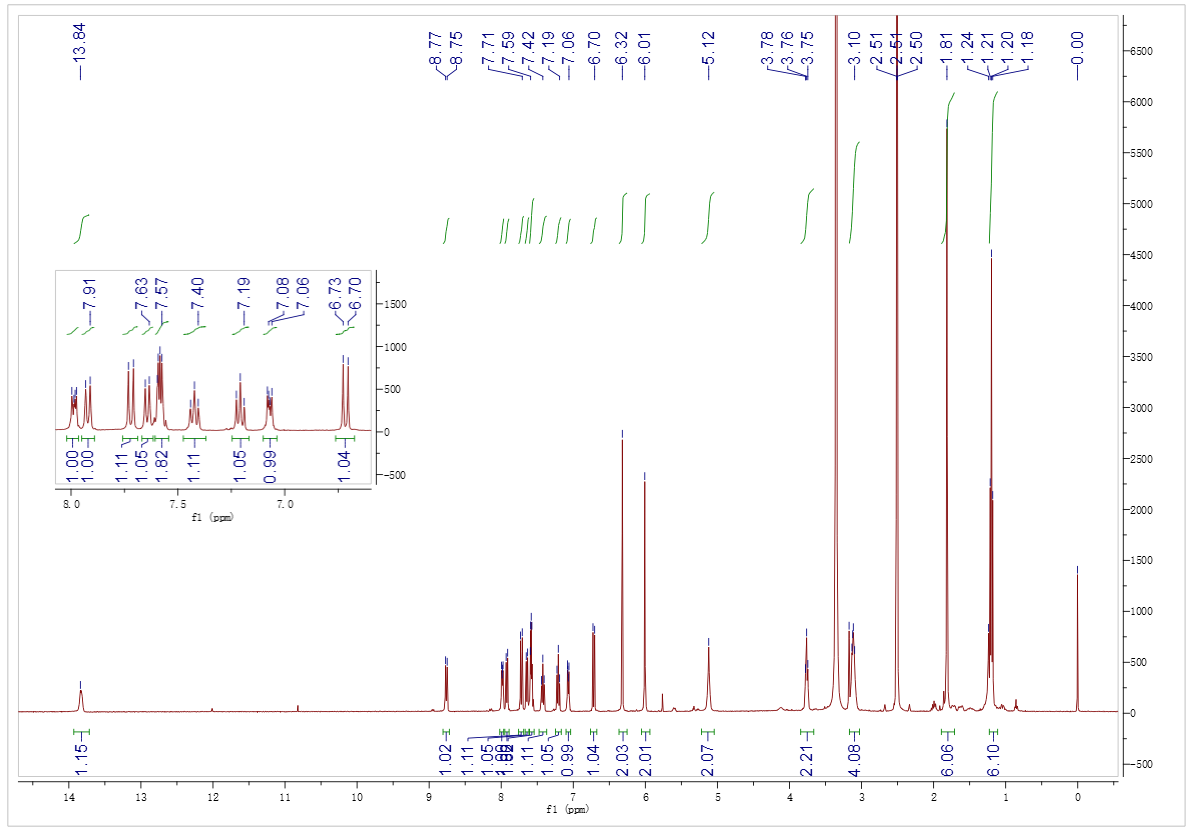


**Figure S1.** ^1^H NMR spectra of compound **L**


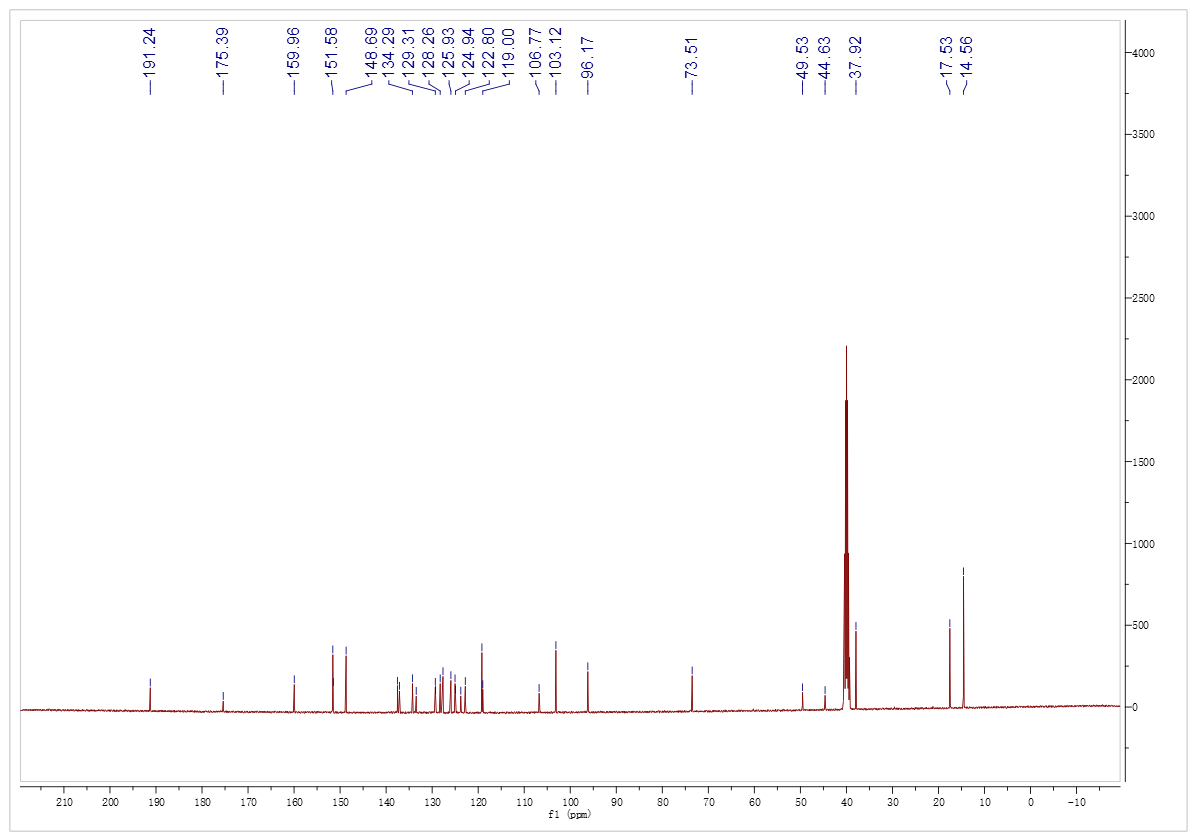


**Figure S2.** ^13^C NMR spectra of compound **L**


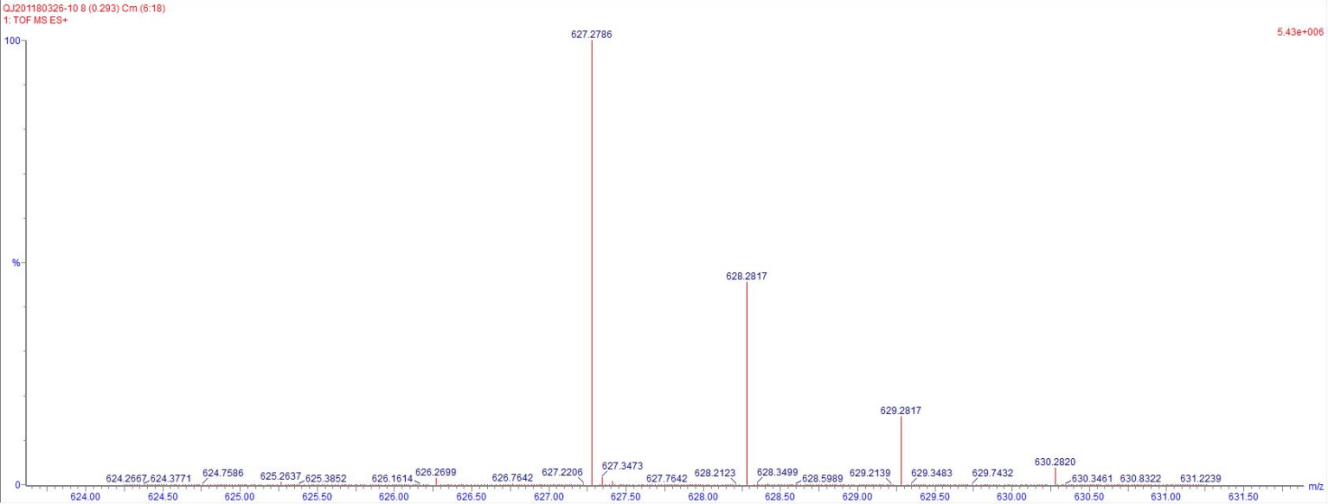


**Figure S3.** ESI-MS spectrum of **L**


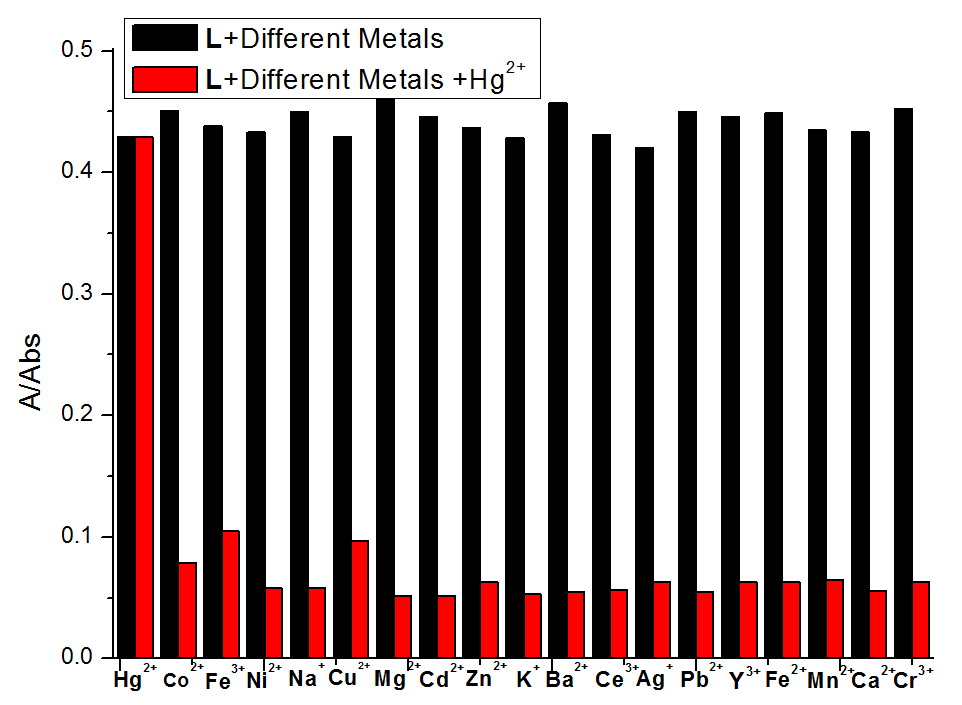


**Figure S4.** Absorption response of **L** (1×10^-5^ M) in HEPES buffer (10 mM, pH 7.4)/CH_3_CN (40:60, V/V) upon addition of respective metal ions, at 526 nm, followed by addition of Hg^2+^.


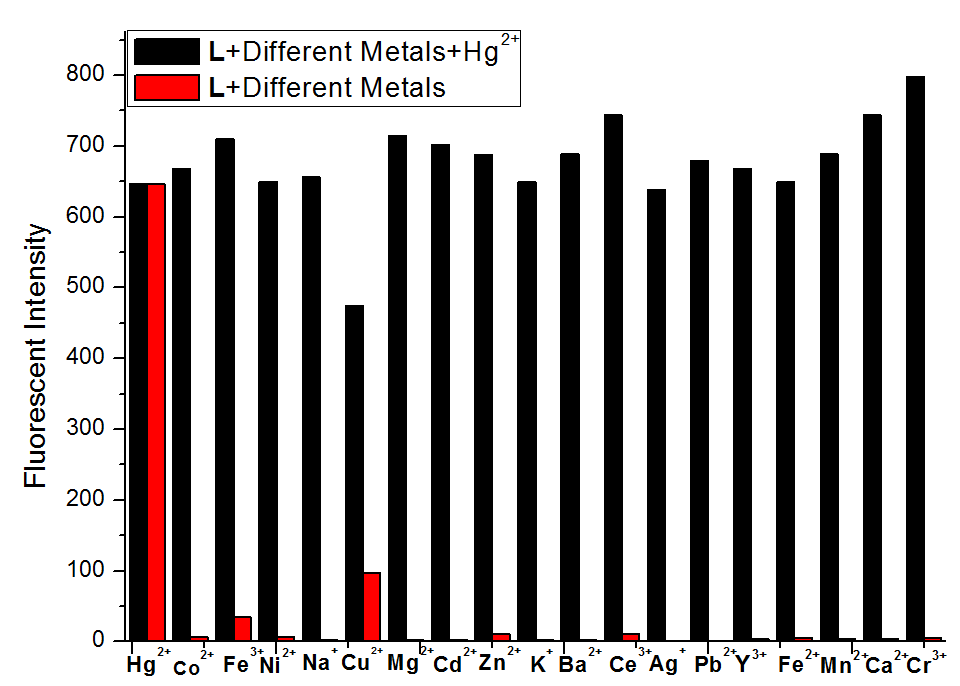


**Figure S5.** Competitive selectivity of **L** (1.0×10^-5^ M) towards Cu^2+^ in the presence of other metal ions in HEPES buffer (10 mM, pH 7.4)/CH_3_CN (40:60, V/V), λ_ex_ = 526 nm, fluorescent intensity at 550 nm.


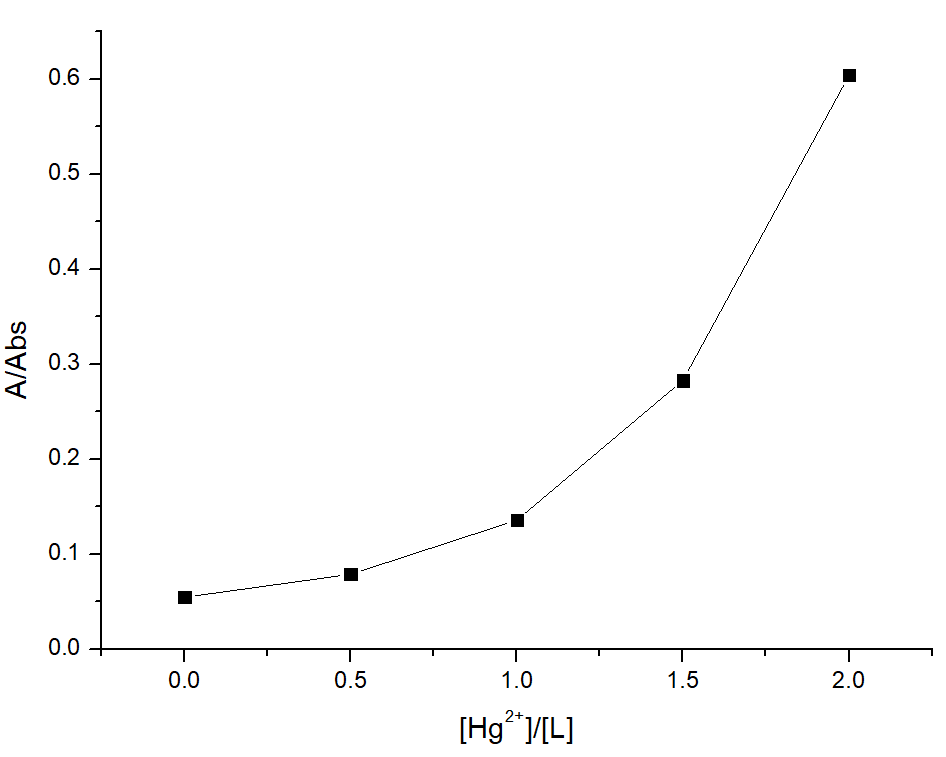


**Figure S6.** Absorption response of **L** (1×10^-5^ M) in HEPES buffer (10 mM, pH 7.4)/CH_3_CN (40:60, V/V), at 526 nm, followed by addition of Hg^2+^. [Hg^2+^]/[**L**]=0, 0.5, 1, 1.5, 2 were separately tested.


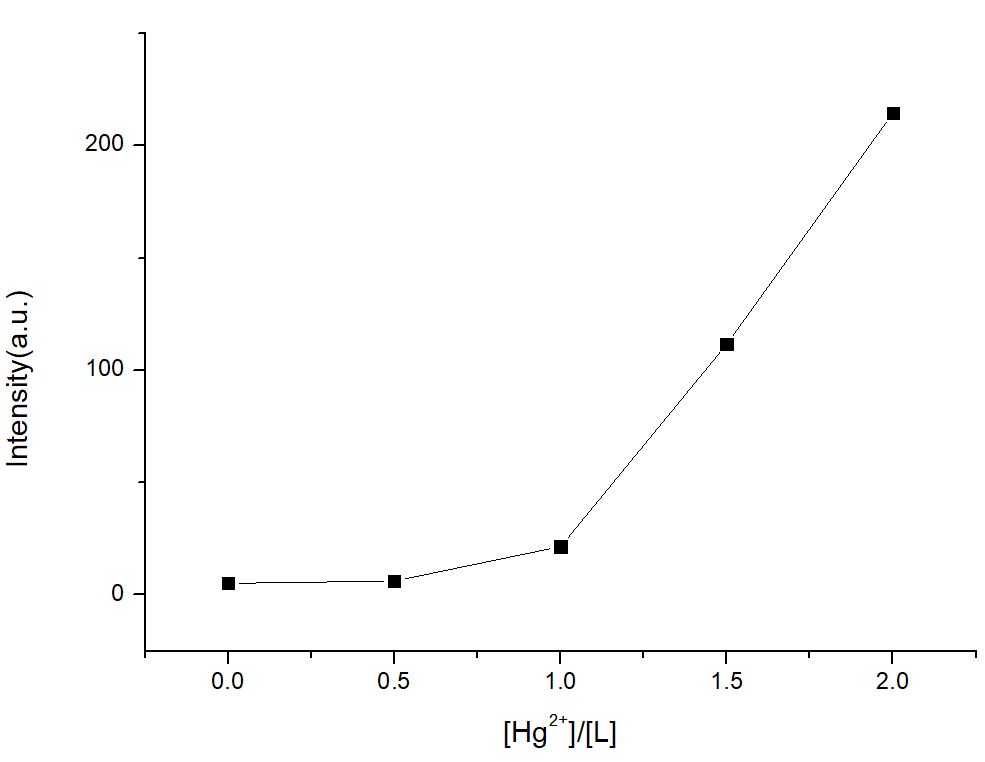


**Figure S7.** Fluorescence spectra of **L** (1×10^-5^ M) in HEPES buffer (10 mM, pH 7.4)/CH_3_CN (40:60, V/V), at 550 nm, followed by addition of Hg^2+^. [Hg^2+^]/[**L**]=0, 0.5, 1, 1.5, 2 were separately tested.


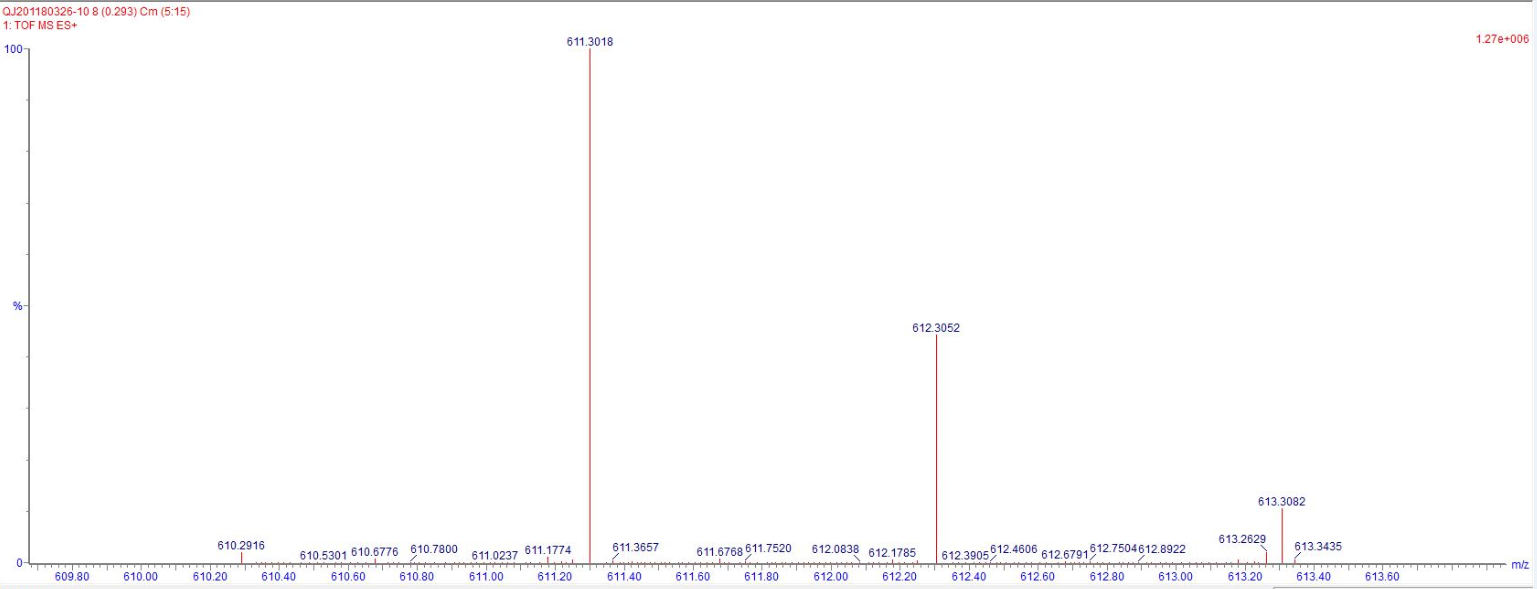


**Figure S8.** ESI-MS spectrum of **LO**


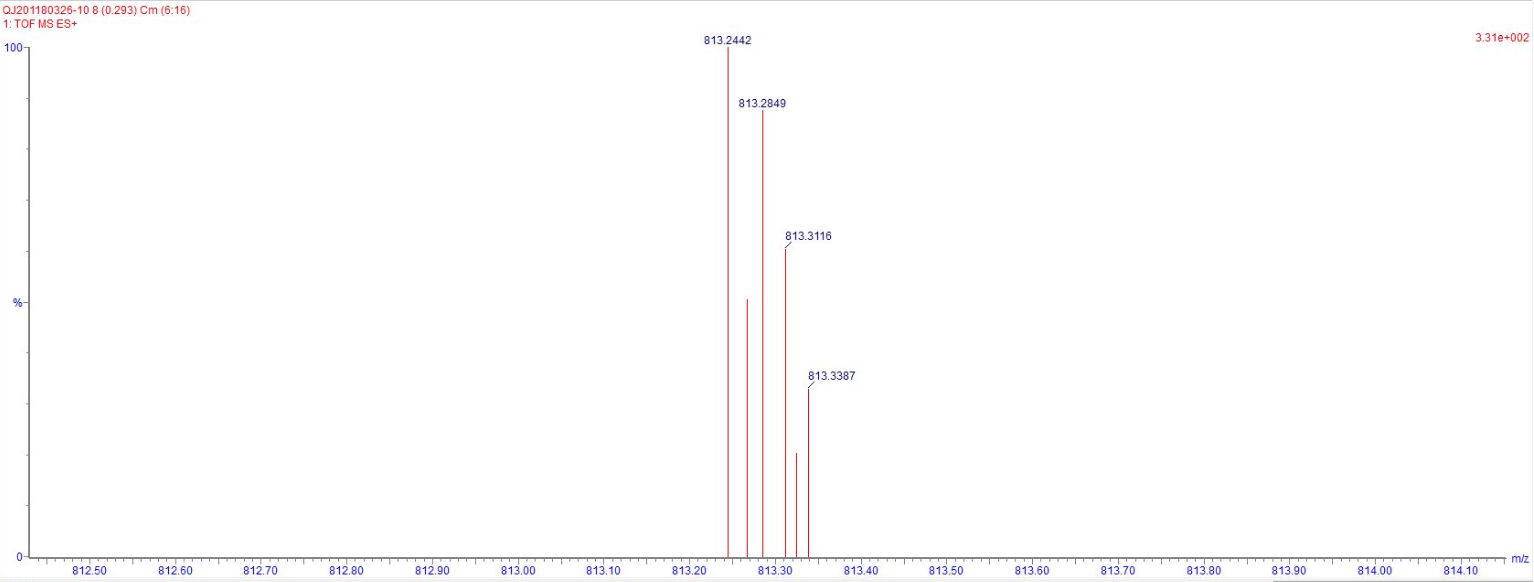


**Figure S9.** ESI-MS spectrum of **LO-Hg**^2+^


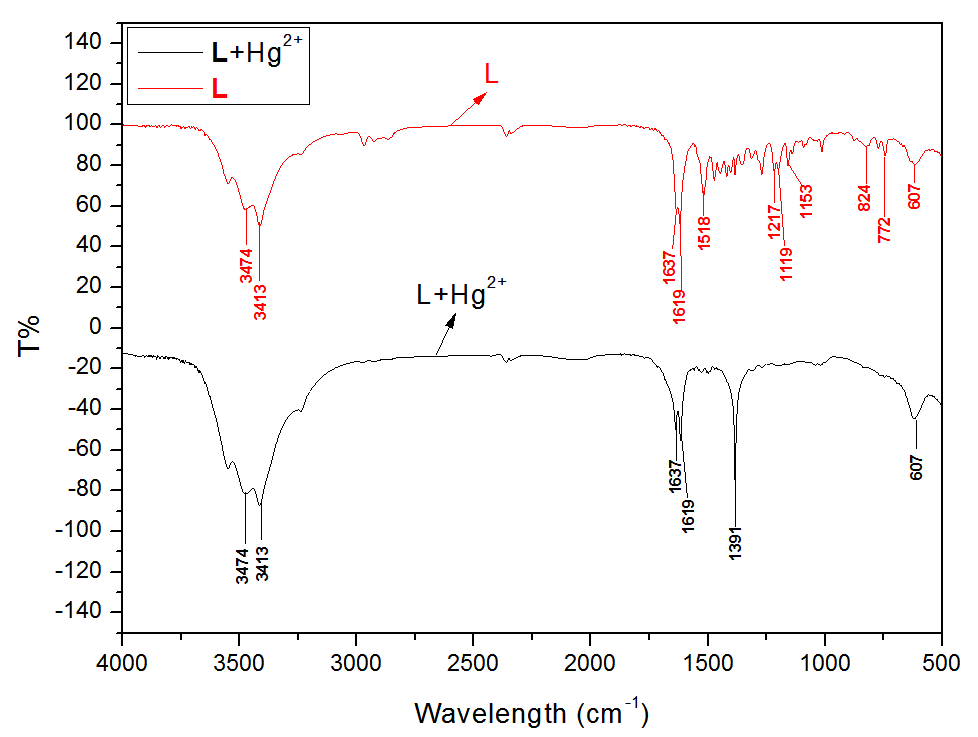


**Figure S10.** FTIR spectra of compound **L** and **L**−Hg^2^**^+^**

(a)


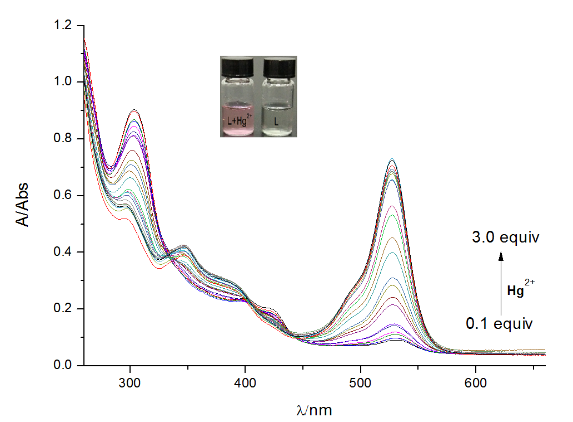


(b)


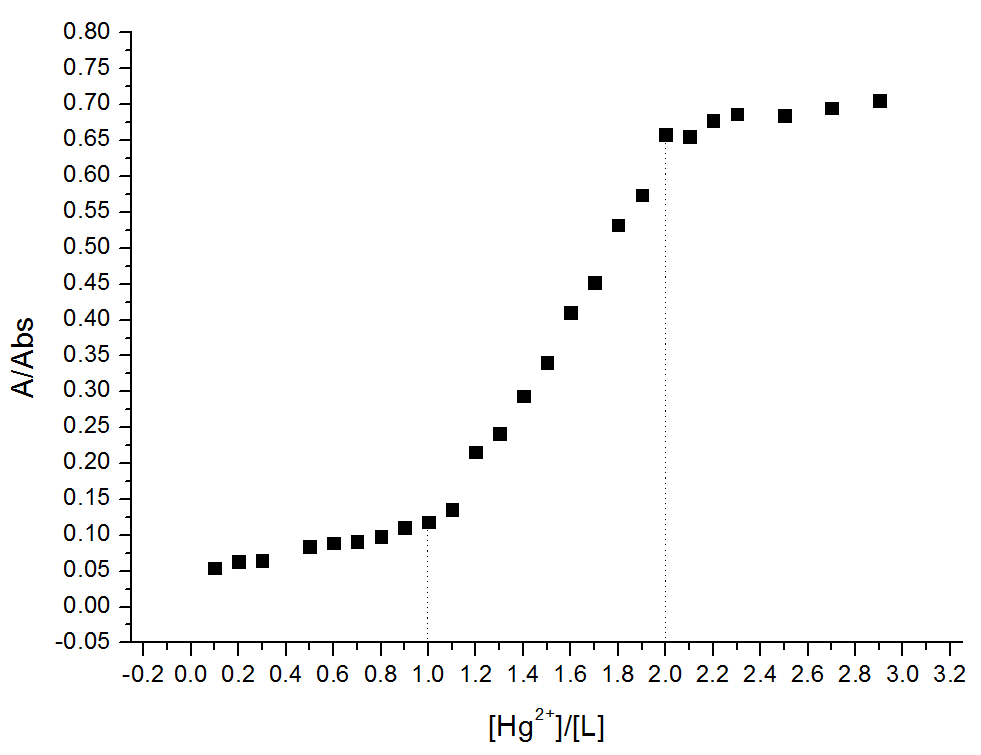


**Figure S11.** (a) Absorption spectra of **L** (1.0×10^-5^ M) in the presence of different concentration of Hg^2+^ (0-3.0 equiv.) in HEPES buffer (10 mM, pH 7.4)/CH_3_CN (40:60, V/V). (b) A plot of absorption depending on the concentration of Hg^2+^ in the range from 0.1 to 3.0 equiv, at 526 nm.


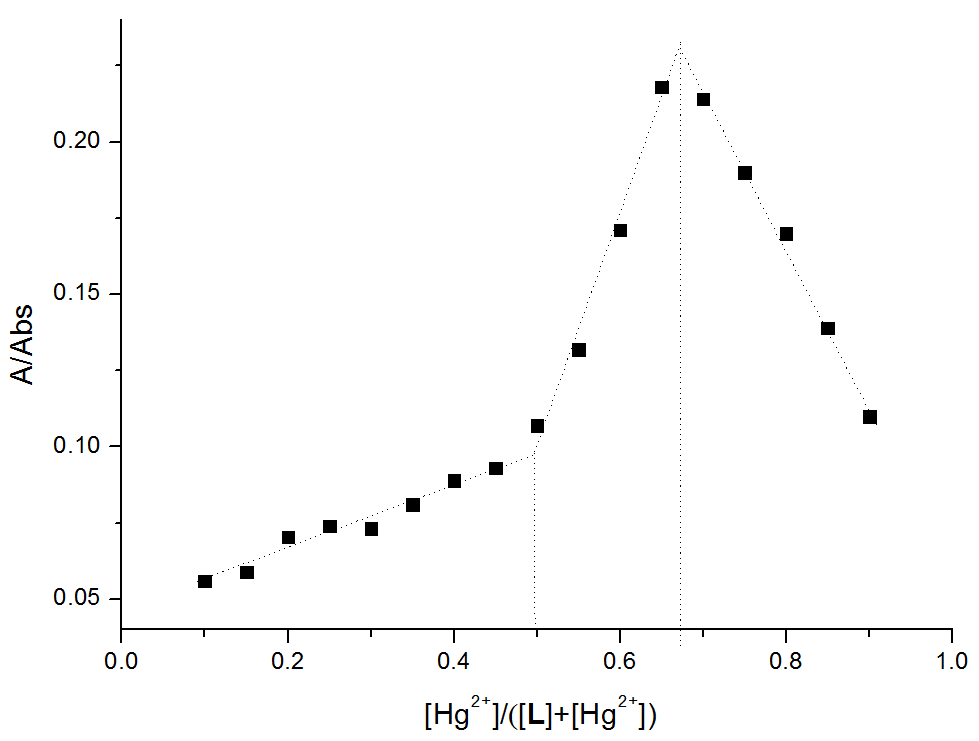


**Figure S12.** Absorption of chemosensors **L** in the presence of Hg^2+^ with different mole ratios of [Hg^2+^]/ ([Hg^2+^+**L**]) at the constant total concentration ([Hg^2+^] + [**L**] = 2.0×10^-5^ M) in HEPES buffer (10 mM, pH 7.4)/CH_3_CN (40:60, V/V). (b) Job’ plot for determination of the binding stoichiometry of chemosensors **L** with Hg^2+^, λ= 526 nm.


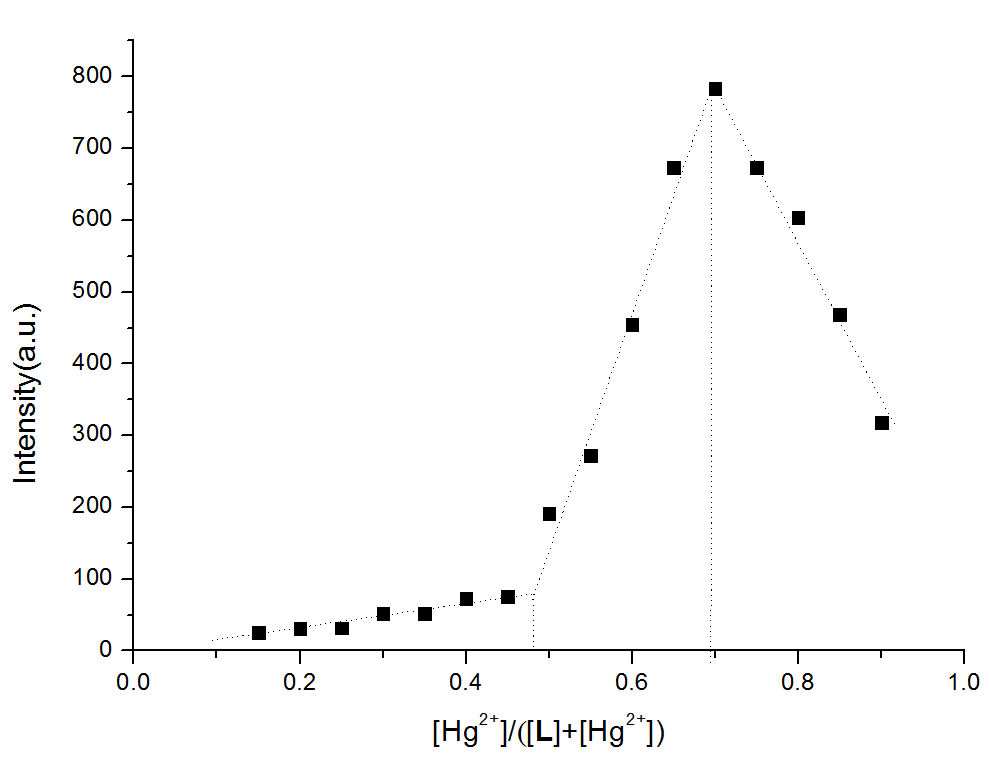


**Figure S13.** (a) Fluorescence spectra of chemosensor **L** in the presence of Hg^2+^ with different mole ratios of [Hg^2+^]/ ([Hg^2+^+**L**]) at the constant total concentration ([Hg^2+^]+[**L**]= 2.0×10^-5^ M) in HEPES buffer (10 mM, pH 7.4)/CH_3_CN (40:60, V/V), λ_ex_= 526 nm, at 550 nm.. (b) Job’ plot for determination of the binding stoichiometry of chemosensors **L** with Hg^2+^.

.


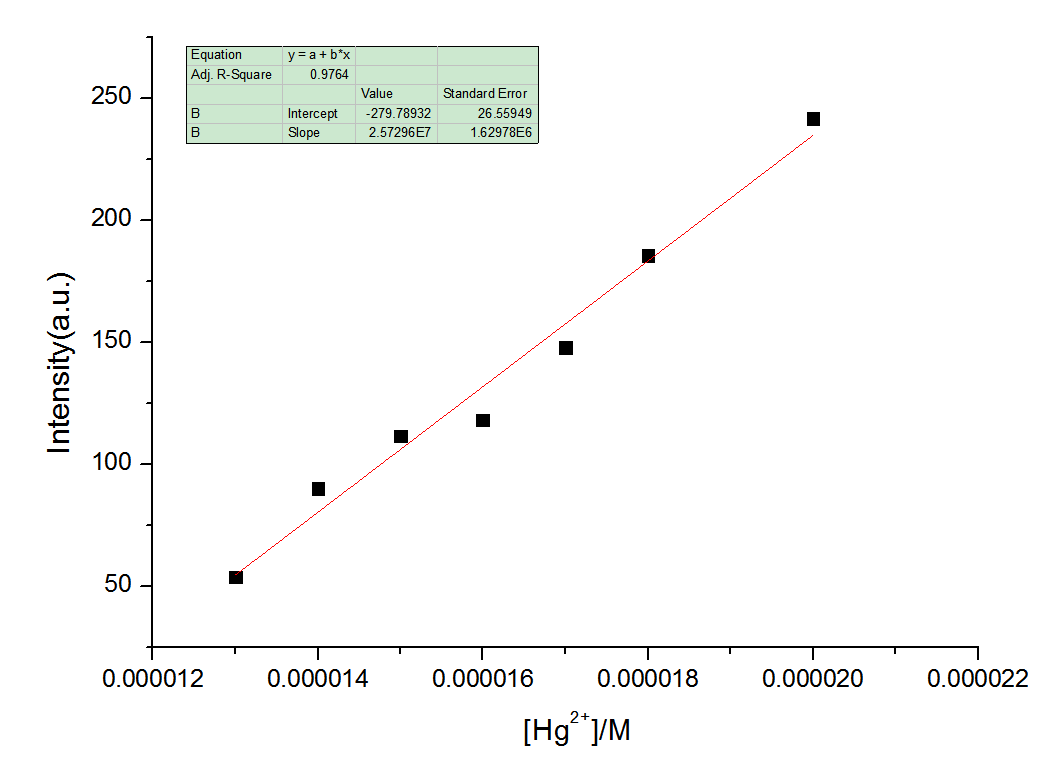


**Figure S14.** Plot of the intensity at 550 nm for a mixture of the sensor **L** (1×10^-5^ M) and Hg^2+^ in HEPES buffer (10 mM, pH 7.4)/CH_3_CN (40:60, V/V), λ_ex_= 526 nm.

S = 2.57296× 10^7^ δ =$\sqrt{\frac{\sum(F_{0}- F_{1})^{2}}{N-1}}$ = 0.01036 (N = 20) K = 3

LOD = K × δ / S = 0.012 × 10^−7^ M

F_0_ is the fluorescence intensity of **L**; F_1_ is the average of the F_0_.

**Table S1**. Fluorescence life time and quantum yield

| **Probes** | **Fluorescence Life Time** | **Fluorescence quantum yield** |
| --- | --- | --- |
| **L**-Hg^2+^ | 4.290913×10^-9^ S | 49.86 % |
